# Amount of Dentifrice and Fluoride Concentration Affect the pH and Inorganic Composition of Dual-Species Biofilms of *Streptococcus mutans* and *Candida albicans*

**DOI:** 10.3390/pharmaceutics16040562

**Published:** 2024-04-20

**Authors:** Caio Sampaio, Alberto Carlos Botazzo Delbem, Thayse Yumi Hosida, Ana Vitória Pereira Fernandes, Bruna do Amaral, Leonardo Antônio de Morais, Douglas Roberto Monteiro, Juliano Pelim Pessan

**Affiliations:** 1 Department of Preventive and Restorative Dentistry, School of Dentistry, São Paulo State University (UNESP), Araçatuba 16015-050, SP, Brazil; alberto.delbem@unesp.br (A.C.B.D.); thayse.hosida@unesp.br (T.Y.H.); ana.vitoria@unesp.br (A.V.P.F.); bruna.amaral@unesp.br (B.d.A.); leonardo.a.morais@unesp.br (L.A.d.M.); douglas@unoeste.br (D.R.M.); juliano.pessan@unesp.br (J.P.P.); 2 Graduate Program in Health Sciences, University of Western São Paulo (UNOESTE), Presidente Prudente 19050-920, SP, Brazil

**Keywords:** dentifrices, fluorides, dental caries, biofilms, *Streptococcus mutans*, *Candida albicans*, child

## Abstract

This work assessed the influence of the amount of dentifrice and fluoride (F) concentration in the product on the pH and inorganic components of *Streptococcus mutans* and *Candida albicans* dual-species biofilms. The biofilms were treated with suspensions of fluoride dentifrices containing 550 or 1100 ppm of F (550 F or 1100 F, respectively) administered at comparable intensities: (i-1) 550 F/0.08 g or 1100 F/0.04 g; (i-2) 550 F/0.16 g or 1100 F/0.08 g; and (i-3) 550 F/0.32 g or 1100 F/0.16 g. A placebo dentifrice (without NaF, 0.32 g) was used as a negative control. After the last treatment, the biofilm pH was measured and the F, calcium (Ca), and phosphorus (P) concentrations were determined. Data were subjected to an ANOVA/Kruskal–Wallis test, and a Student–Newman–Keuls test. The highest biofilm pH and F concentrations (biomass and fluid) were observed for 1100 F at i-3. Overall, 1100 F resulted in F levels similar to 550 F for i-1 and i-2. In addition, 550 F applied at i-2 and i-3 led to higher F in the biomass/fluid compared to 1100 F applied at i-1 and i-2, respectively. In biomass, the lowest Ca concentrations were observed for 1100 F at i-3. The conclusion drawn is that the treatment intensity holds greater significance as a parameter compared to the concentration of F or the amount of dentifrice when considered individually.

## 1. Introduction

Biofilms are complex microbial communities that are extensively organized, adhering to both living and non-living surfaces, and surrounded by an extracellular matrix produced by the constituent cells [1]. This matrix consists of extracellular material, mainly made up of polysaccharides, proteins, nucleic acids, and lipids, which provide the biofilm with mechanical stability and adhesion to surfaces, in addition to acting as an external digestive system, keeping extracellular enzymes close to the cells, and allowing them to metabolize dissolved colloidal substances and solid biopolymers [2]. In addition, the imbalance of inorganic components (i.e., calcium and phosphate) between the tooth surface and biofilm is an important factor in determining the cariogenic process [3,4,5]. Dental caries is a disease that is influenced by biofilm (dental plaque), modulated by dietary factors, multifactorial, untransmissible, and dynamic, resulting in the progressive mineral loss of dental hard tissues, further leading to tooth loss [6]. In summary, two factors are crucial for caries onset and progression: a sugar-rich diet and the presence of biofilm [6,7,8].

Considering the dynamics of dental caries, the widespread use of fluoride dentifrices has been attributed to one important reason for the decline in the prevalence and incidence of dental caries worldwide [9]. This occurs because, unlike other forms of fluoride (F) administration where their effects are limited to the chemical activity of F [10], fluoride dentifrices combine the mechanical removal or disruption of dental biofilm with the therapeutic effects of F on the processes of demineralization and remineralization of dental tissues. These effects include reducing enamel solubility in an acidic environment, inhibiting the acquisition and utilization of glucose by acidogenic bacteria, and promoting enamel remineralization [11,12,13]. Nonetheless, despite the important role that F plays in caries control, an excessive F intake (especially from a dentifrice) during the formation of permanent teeth (i.e., particularly at 2–3 years of age) can lead to dental fluorosis [14]. Therefore, some alternatives have been proposed to minimize the F intake from these sources, including the use of dentifrices with low F concentrations or decreasing the amount of product applied to the toothbrush according to each age group. However, in addition to the fact that scientific evidence for these recommendations still requires further investigation, a wide variation in how parents and children understand and interpret these recommendations has been noted, given the variety of terminology used by professionals/societies, which include “small quantity”, a “smear”, a “pea-size amount”, a “rice grain”, and “transverse technique” [15,16,17,18]. 

The influence of the amount of dentifrice used and brushing time on salivary F levels, the F incorporation into enamel, and the remineralization of incipient caries lesions has been studied in an in situ model [19], according to which the authors demonstrated a direct relationship between the studied variables (time and amount of dentifrice) and F incorporation into enamel, saliva uptake, and enamel remineralization. From this perspective, prospective studies demonstrated no difference in caries progression [20] and in biofilm F uptake [21] in individuals using different quantities of dentifrices during toothbrushing. In addition, Den Besten et al. [22] demonstrated a three-fold increase in the F concentration in the saliva of children using 1 g of dentifrice during brushing compared to children using 0.25 g. Considering that the clinical effectiveness of F dentifrices is directly proportional to the intraoral fluoride uptake [20,23], recent studies have evaluated the impact of using different amounts of dentifrice on the salivary F levels in children using a standard F dentifrice (1000–1100 µg F/g) and a dentifrice with a low F concentration (500–550 µg F/g), in which it was observed in a clinical trial with 8–10 year-old children that brushing with a dentifrice containing 550 ppm of F using the smear technique resulted in a higher F concentration in the saliva compared to a conventional dentifrice using a pea-sized amount, suggesting that using small amounts of a conventional dentifrice may not be as effective as using a dentifrice with a low F concentration applied using the smear technique [24]. However, a recent trial with 2–3-year-old children found that brushing with a 1100 ppm F dentifrice, applied at 0.16 g, resulted in significantly higher salivary F concentrations than a dentifrice with 550 ppm of F, applied at 0.32 g [25].

Despite the aforementioned trends regarding salivary F concentrations in children after using different combinations of dentifrice amounts and F concentrations in the product, information about the effect of these variables on biofilms is still scarce. Some studies have reported that F concentration directly affects the virulence and composition of cariogenic biofilms formed in vitro [26,27,28,29]. However, to date, no study has evaluated the influence of treatment intensity (i.e., the amount of dentifrice used × the F concentration of the product) on the inorganic composition and biofilm pH. Therefore, considering the important role that dental biofilm plays in the cariogenic process, combined with the scarcity of information about the influence of treatment intensity on biofilm composition, this study aimed to evaluate the effect of dentifrice slurries containing 0, 550, or 1100 ppm of F, applied in different amounts, on the pH and concentrations of F, phosphorus (P), and calcium (Ca) in dual-species biofilms (fluid and biomass) of *Streptococcus mutans* and *Candida albicans*. The null hypothesis of the study is that the F concentration of the dentifrice or the amount of dentifrice used during treatment and the F concentration in the product do not influence the inorganic composition and pH of the analyzed biofilm.

## 2. Materials and Methods

### 2.1. Preparation of Artificial Saliva

The biofilms were grown in sucrose-supplemented artificial saliva containing 2 g of yeast extract, 5 g of bacteriological peptone, 4 g of sucrose, 1 g of mucin, 0.35 g of sodium chloride, 0.2 g of calcium chloride, and 0.2 g of potassium chloride in 1 L of deionized water. The pH of the solution was set with 1M sodium hydroxide to 6.8 [30,31]. All reagents were purchased from Sigma-Aldrich (St Louis, MO, USA).

### 2.2. Strains of Microorganisms, Growth Conditions

The experiments utilized strains obtained from the American Type Culture Collection (ATCC). *C. albicans* ATCC 10231 cultures were seeded on a Sabouraud Dextrose Agar (ASD; Difco, Le Pont de Claix, France) and incubated at 37 °C for 24 h. Meanwhile, *S. mutans* ATCC 25175 cultures were seeded on a Brain Heart Infusion Agar (BHI Agar; Difco, Le Pont de Claix, France) and incubated in a 5% CO_2_ environment at 37 °C for 24 h [31]. To prepare the *C. albicans* culture, a loopful of cells from the Sabouraud Dextrose Agar (ASD) plates was suspended in 10 mL of Sabouraud Dextrose broth (Difco; Le Pont de Claix, France). The culture was then incubated at 37 °C overnight with shaking at 120 rpm. Colonies of *S. mutans* were suspended in 10 mL of BHI broth (Difco) and incubated statically overnight in 5% CO_2_ at 37 °C. After the incubation period, fungal and bacterial cells were centrifuged at 8000 rpm for 5 min at 15 °C, and the cell pellets were washed twice with 10 mL of 0.85% sodium chloride (Sigma-Aldrich; St. Louis, MO, USA). The concentration of the fungal cells was set at 10^7^ cells/mL in artificial saliva using a Neubauer chamber and an optical microscope, while the bacterial cell quantity was spectrophotometrically adjusted to a concentration of 10^8^ cells/mL in the artificial saliva. The growth media were replenished every 24 h [31]. The biofilms were formed at the bottom of 6-well microtiter plates (CostarVR #3516, Corning Inc., Corning, NY, USA) by adding 4 mL of the suspension to the wells. 

### 2.3. Treatment of the Biofilms

The biofilms were treated after 72, 78, and 96 h of formation (totaling 3 treatments) [31] with dentifrices of different F concentrations (0, 550, or 1100 ppm of F, as NaF), applied in different quantities according to 3 different intensities (Table 1) [25]. The clinical reference for the quantities of dentifrice used was based on clinical recommendations by scientific societies [32,33], resulting in 3 quantities, 0.08, 0.16, and 0.32 g, representing a “rice grain”, a “pea-size amount”, and the “transverse technique”, respectively [25]. The experimental groups were formulated with the aim of producing direct comparisons regarding the final F concentrations, considering the amount of dentifrice applied to the brush and the F concentration in the toothpaste (i.e., 0.08 g of 550 ppm F dentifrice × 0.04 g of 1100 ppm F dentifrice; 0.16 g of 550 ppm F dentifrice × 0.08 g of 1100 ppm F dentifrice; and 0.32 g of 550 ppm F dentifrice × 0.16 g of 1100 ppm F dentifrice). A F-free (0 ppm F) dentifrice administered at 0.32 g was tested as the negative control [25]. 

The treatments were performed by gently pipetting 4 mL of the slurry onto the wells, aspirating it back after 1 min (simulating an average brushing time [24,25]), and washing the biofilms twice with 4 mL of 0.85% sodium chloride by gently pipetting it onto the wells and immediately removing it back from the wells in order to remove the remaining content of the dentifrice [31].

### 2.4. Determination of pH and Analysis of F, P, and Ca in Biofilm Fluid and Biomass

After the last treatment, the biofilms were scraped from the wells of the microtiter plates using a cell scraper (Kasvi, São José dos Pinhais, Brazil). The scraped biofilms were then transferred to microtubes (MCT-200-C-Axygen; Glendale, AZ, USA) using a pipette. The pH of the biofilms was measured using a pH electrode, specifically the PHR-146 micro combination pH electrode from Fisher Scientific (Hampton, VA, USA), previously calibrated with pH 4 and 7 standards [31]. Next, the microtubes containing the scraped biofilms were centrifuged at 15,267× *g* for 5 min at 4 °C. This allowed the biofilm fluid to be collected [31].

To analyze the F concentrations, an ion-selective electrode (Orion 9409 BN; Thermo Scientific, Waltham, MA, USA) and a reference electrode (Orion 900100; Thermo Scientific, Waltham, MA, USA) were used. Both electrodes were coupled to a potentiometer from Orion-Thermo Scientific (Waltham, MA, USA). Calibration curves for F analysis in the biofilm fluid were created using standards of 0.09, 0.18, 0.36, 0.72, and 1.44 μg F/mL (for biofilms treated with F-free solutions) and 6.25, 12.5, 25, 50, and 100 μg F/mL (for biofilms treated with solutions containing F). A total ionic strength adjustment buffer (TISAB II) was used in a 1:1 ratio with the samples under the same conditions [31].

The Ca concentrations were determined via spectrophotometry using a plate reader (EON spectrophotometer from EON, Biotek, Winooski, VT, USA) at a 650 nm wavelength, as detailed by Vogel, Chow, and Brown [34]. Briefly, a duplicate aliquot of 5 μL for both standards and samples was mixed with 50 μL of Arsenazo III (Sigma-Aldrich; St Louis, MO, USA), which is a colorimetric reagent. Additionally, 50 μL of deionized water was added to the mixture. The mixture was then shaken for 60 s in a microplate reader to facilitate the reaction between the sample and Arsenazo III. After shaking, the resulting absorbances were obtained. The concentrations of total P were determined using the method proposed by Fiske and Subbarow [35]. In summary, 100 µL of the samples, 50 µL of molybdate (Sigma-Aldrich; St Louis, MO, USA), and 20 μL of a reductive reagent were combined. The mixture was then read at a wavelength of 660 nm in a microplate reader.

For the analysis of F, Ca, and P in the total biofilm biomass, hydrochloric acid (0.5 mol/L; Sigma-Aldrich; St Louis, MO, USA) was added to the microtubes with the biofilms at a ratio of 0.5 mL/10.0 mg of wet weight of the plate [36] and homogenized. The resulting mixture was kept at room temperature with constant agitation (120 rpm) for 3 h and then centrifuged (11,000× *g*; for 1 min) [37]. Then, 400 μL of the liquid was removed, and the same volume of 0.5 mol/L sodium hydroxide was added. The F content in the biofilm biomass was analyzed, as described above for the biofilm fluid, using standards containing 0.09, 0.18, 0.36, 0.72, and 1.44 μg F/mL (for biofilms treated with F-free solutions) and 0.8, 1.6, 3.2, 6.4, and 12.8 μg F/mL (for biofilms treated with solutions containing F) [31]. The Ca and P components in the biomass were analyzed following the method described above for the biofilm fluid [34,35].

### 2.5. Statistical Analysis

Data on Ca and P concentrations in the biofilm fluid passed the normality (Shapiro–Wilk) test (*p* = 0.339; *p* = 0.205, respectively) and the equal variance test (*p* = 0.384; *p* = 0.133, respectively) and were subjected to an ANOVA and Student–Newman–Keuls test. Data on the F, Ca, and P concentrations in the biofilm biomass and F in the biofilm fluid did not pass the normality (Shapiro–Wilk) test (*p* = 0.050; *p* = 0.050; *p* = 0.050; *p* = 0.050, respectively) and were analyzed using the Kruskal–Wallis test and the Student–Newman–Keuls test. Data on the biofilm pH passed the normality test (Shapiro–Wilk) (*p* = 0.527) but did not pass the equal variance test (*p* = 0.050) and were analyzed using the Kruskal–Wallis test and the Student–Newman–Keuls test. A statistical analysis was conducted using SigmaPlot 12.0 software (San Jose, CA, USA), adopting *p* < 0.05.

## 3. Results

The mean (standard deviation) ionic and total F concentrations in the dentifrices were, respectively, 539.4 (44.1) and 597.2 (21.1) ppm of F for the 550 ppm F dentifrice and 1149.7 (24.1) and 1174.3 (35.4) ppm of F for the 1100 ppm F dentifrice. For the placebo dentifrice (i.e., without F), the mean (standard deviation) ionic and total F concentrations were, respectively, 11.9 (2.1) and 12.7 (2.8) ppm of F.

**Figure 1:**
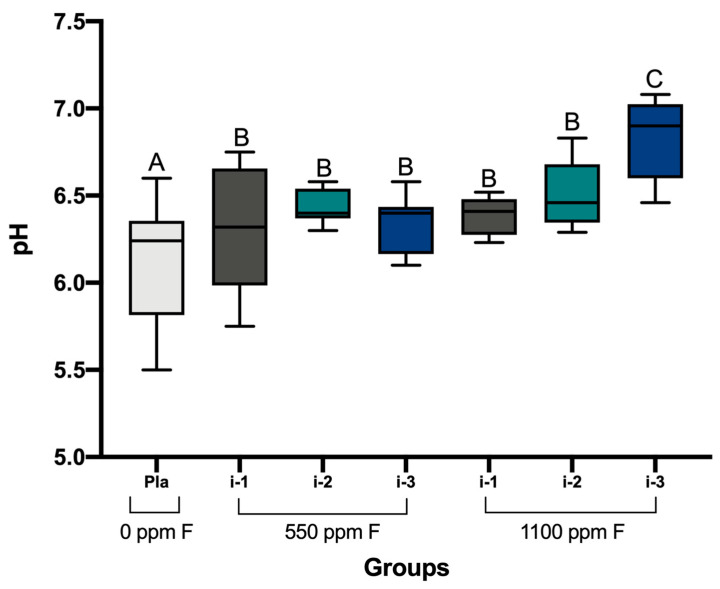
Biofilm pH after treatments according to the experimental groups. Different letters denote significant differences among the groups (Student–Newman–Keuls test, *p* < 0.05, *n* = 9). F = fluoride; Pla = placebo (fluoride-free).

Regarding the biofilm pH, significant differences were observed among the treatments (*p* ≤ 0.001). All the biofilms treated with F-containing dentifrices, regardless of the treatment intensity, had significantly higher pH values than the placebo. The biofilms treated with 1100 ppm of F at i-3 showed the highest pH values (Figure 1).

**Figure 2:**
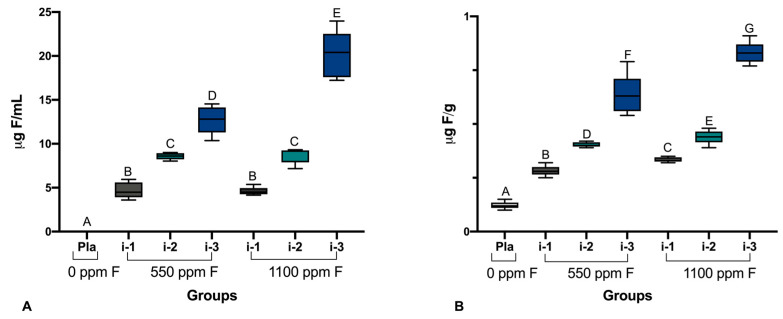
Fluoride concentrations in the biofilm fluid (**A**) and biomass (**B**). Different letters denote significant differences among the groups (Student–Newman–Keuls test, *p* < 0.05, *n* = 9). F = fluoride; Pla = placebo (fluoride-free).

Regarding the F concentrations in the biofilm fluid and biomass, the statistical analysis indicated a significant difference among the groups (*p* ≤ 0.001). The F concentrations in both the biofilm biomass and fluid increased proportionally with the F concentration in the dentifrice used and the amount of dentifrice administered. In general, 1100 ppm of F resulted in similar F concentrations to 550 ppm of F for i-1 and i-2. However, higher F concentrations were observed for the i-3 treatment with 1100 ppm of F. Additionally, 550 ppm of F applied at higher intensities (i-2 and i-3) led to significantly higher F concentrations in the biomass and fluid compared to 1100 ppm of F administered at lower intensities (i-1 and i-2, respectively) (Figure 2).

**Figure 3:**
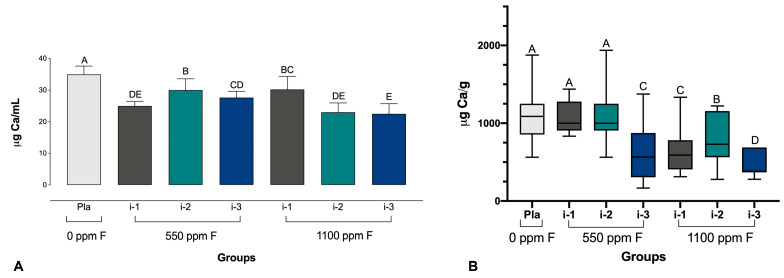
Calcium concentrations in the fluid (**A**) and biomass (**B**) of biofilms. Different letters denote significant differences between the groups (Student–Newman–Keuls test, *p* < 0.05, *n* = 9). Ca = calcium; Pla = placebo (fluoride-free).

Regarding the F concentrations in the biofilm fluid and biomass, the statistical analysis indicated a significant difference among the groups (*p* ≤ 0.001). It was observed that in the biofilm fluid, all the groups treated with fluoride dentifrices had significantly lower Ca values compared to the placebo group. In the biofilm biomass, it was possible to observe that the group with the lowest level of Ca was the 1100 ppm F group at intensity 3 (Figure 3).

**Figure 4:**
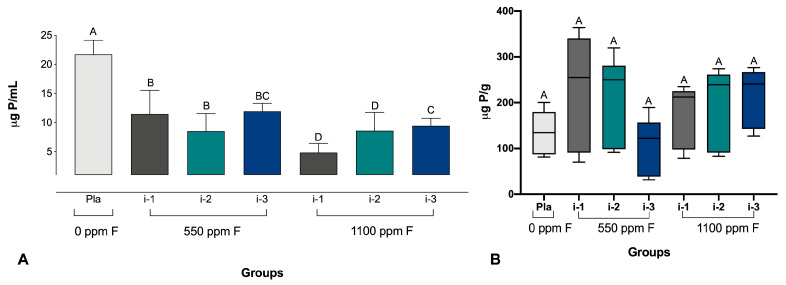
Phosphorus concentrations in the biofilm fluid (**A**) and biomass (**B**). Different letters denote significant differences among the groups (Student–Newman–Keuls test, *p* < 0.05, *n* = 9). P = phosphorus; Pla = placebo.

As for the P in the biofilm biomass and fluid, the statistical analysis demonstrated, respectively, *p* = 0.033 and *p* ≤ 0.001, indicating significant differences among the groups. It was observed that the P concentrations in the fluid of the biofilms treated with fluoride dentifrices were significantly lower than those of the biofilms treated with the placebo dentifrice, with no significant differences, however, between the groups treated with dentifrices within the same F concentrations (Figure 4).

## 4. Discussion

The scarce evidence regarding the influence of the treatment intensity (i.e., the amount of dentifrice used × the F concentration of the product) on outcomes related to dental caries prompted the development of this study. It was demonstrated that the inorganic composition and pH of cariogenic-related biofilms (*S. mutans* and *C. albicans*) varied depending on the treatments with dentifrices containing different F concentrations, administered in different quantities, leading to rejection of the study’s null hypothesis.

In this study, dentifrices applied in smaller quantities, regardless of the F concentration in the product, resulted in lower F concentrations in the biofilm (fluid and biomass). Furthermore, the F concentrations, especially in the biofilm biomass, were significantly higher for the groups treated with 1100 ppm of F compared to 550 ppm of F within the same treatment intensities. These trends corroborate those found in a previous study, which demonstrated that a dentifrice with 1100 ppm of F resulted in higher concentrations of F in the saliva of toddlers when compared to a dentifrice with 550 ppm of F within the same treatment intensity [25]. At the time, among the possible hypotheses, the authors considered that dentifrice ingestion during brushing, as well as the dilution and stability of the product, could have influenced the results. Given the absence of the variable “ingestion” due to the in vitro nature of this study, it is possible that the latter influenced the patterns found. It is reasonable to consider that more consistent slurries obtained through treatment intensities containing larger amounts of dentifrice may have presented greater difficulty in penetrating the biofilms, thus hindering F incorporation into the biofilm compartments. 

It has been demonstrated that during short periods of exposure, which includes toothbrushing conditions, certain molecules can only penetrate the outer third of the biofilm layer. The outer region of the biofilm has a higher biomass density compared to the inner layers. This higher density hinders the penetration of external materials into the biofilm. This explains why certain substances, such as nutritional and metabolic waste products, minerals from the tooth surface, and therapeutic products targeted at influencing dental plaque, have limited access to the deeper layers of the biofilm [38]. Furthermore, the structure of biofilms, particularly in the outer layers, is resistant to both mechanical and chemical attacks. However, the presence of detergents can cause significant changes in the biofilm structure. This can have a significant impact on the transfer of materials, such as dentifrices, into and out of the biofilm, especially during the delivery process [39]. In summary, the outer region of the biofilm restricts the penetration of external substances, and the structure of the biofilm can be altered by detergents, which can affect the transfer of materials into and out of the biofilm. In addition to this assumption, it is possible that the same F concentration disposed of in a larger amount of dentifrice may have favored interactions with other components of the dentifrice, impairing its penetration into the biofilms, which needs to be further confirmed in future appropriate models.

Interestingly, the aforementioned patterns regarding F concentrations were reflected in the pH values of the biofilms after the treatments, as 1100 ppm of F at i-3 resulted in the highest pH values. F has been previously demonstrated to decrease the acid-producing capacity of *S. mutans* biofilms [12,13], which was further confirmed in recent data [31]. In summary, HF penetrates the bacterial cell membrane, dissociating into H^+^ and F^−^, which are more alkaline than the external environment. Thus, F^−^ has an effect on glycolytic enzymes, leading to a reduction in acid production from glycolysis, further affecting microbial viability and metabolism [12,13]. This study was the first to investigate the influence of the amount of dentifrice used, as well as the F concentration of the product, on the pH of cariogenic-related biofilms.

Another finding worth mentioning is regarding the concentrations of Ca in the biofilm biomass. Biofilms from the 1100 ppm F group at the highest treatment intensity had significantly lower values of this component in contrast to previous in vivo studies [40]. Although the reasons for these discrepancies are not clear, it is important to note the marked difference in study designs. While the mentioned in vivo studies include variables such as rinsing and salivary flow, in the present work, the biofilms underwent identical rinsing conditions with sodium chloride and did not have an active salivary flow; therefore, it is reasonable to assume that these aspects may have substantially influenced the Ca concentrations found in the mentioned studies as well as in the present study.

Despite the above-mentioned findings regarding the pH and F and Ca concentrations in the biofilms, a less defined pattern was observed for the levels of P in the biofilms (fluid and biomass). Although the reasons for these data are not clear, it can be considered that possible interactions between the components of the dentifrices and the culture medium (i.e., artificial saliva) may have influenced this scenario, which is why lower *p* values were observed for the groups treated with fluoride dentifrices, regardless of their F concentration. Therefore, future studies addressing this variable should be conducted in order to clarify such patterns.

Although the trends found in this study provided relevant information regarding the inorganic composition and pH of biofilms after the use of different treatment intensities, the research protocol employed in this study has some limitations. Firstly, the in vitro nature of this work, which is very far from in vivo, restricts the extrapolation of the results to clinical conditions. In addition, it is important to note that the absence of a mineral substrate complicates making more advanced extrapolations on the topic. Further investigations including dental enamel could provide relevant information about the ion exchange of biofilms with the oral environment and the mineral substrate, considering different protocols for using fluoride dentifrices, including varying quantities of the product and using different concentrations of F. Another important aspect to mention is the lack of different washing/rinsing protocols and the frequency of the treatments, as they are important factors related to F uptake [41,42,43,44]. These variables should be considered in future protocols. Lastly, it is important to acknowledge that the wide variety of toothpaste compositions, including the addition of other active ingredients, may lead to different outcomes compared to those found in the present study [45]. It is important to note as well that this study adopted the use of NaF as this is most commonly used for children, and the use of a different F salt could also lead to different results, considering their different modes of action [46]. Despite these limitations, the strictly controlled conditions adopted in this protocol were important to test a hypothesis (proof of concept) based on empirical observations (i.e., dilution of toothpaste (and fluoride) in saliva and subsequent reduction in the anticaries effect), which had not been previously assessed under controlled experimental conditions.

## 5. Conclusions

Based on the information presented above, the conclusion drawn is that the treatment intensity holds greater significance as a parameter compared to the concentration of F or the amount of dentifrice when considered individually. The findings presented in this study favor a better understanding of this topic and add relevant information to the body of evidence on this issue, which can further contribute to the development of regimes for the use of fluoride dentifrices for children.

## Figures and Tables

**Table 1:** Distribution of study groups according to the amount of dentifrice used and fluoride concentrations of the dentifrices.

Intensities	Amount of Dentifrice	Fluoride Concentration in the Dentifrice
i-1	0.08 g	550 ppm F
i-2	0.16 g	
i-3	0.32 g	
i-1	0.04 g	1100 ppm F
i-2	0.08 g	
i-3	0.16 g	
negative control	0.32 g	Placebo (fluoride-free; 0 ppm F)

## Data Availability

The data presented in this study are available on request from the corresponding author.
